# Study of The Reaction Mechanism to Produce Nanocellulose-Graft-Chitosan Polymer

**DOI:** 10.3390/nano8110883

**Published:** 2018-10-30

**Authors:** Jose Luis Sanchez-Salvador, Ana Balea, M. Concepcion Monte, Angeles Blanco, Carlos Negro

**Affiliations:** Department of Chemical Engineering and Materials, Universidad Complutense de Madrid, Av. Complutense s/n, 28040 Madrid, Spain; josanc03@ucm.es (J.L.S.-S.); anabalea@ucm.es (A.B.); cmonte@ucm.es (M.C.M.); cnegro@ucm.es (C.N.)

**Keywords:** cellulose nanofibers, cellulose microfibers, chitosan, grafting, reaction mechanism, nanocellulose-graft-chitosan, water-based inks

## Abstract

Cellulose and chitin are the most abundant polymeric materials in nature, capable of replacing conventional synthetic polymers. From them, cellulose nano/microfibers (CNFs/CMFs) and chitosan are obtained. Both polymers have been used separately in graft copolymerization but there are not many studies on the use of cellulose and chitosan together as copolymers and the reaction mechanism is unknown. In this work, the reaction mechanism to produce nano/microcellulose-graft-chitosan polymer has been studied. Recycled cellulose pulp was used, with and without a 2,2,6,6-tetramethylpiperidin-1-oxyl-radical (TEMPO)-mediated oxidation pretreatment, to produce CNFs and CMFs, respectively. For chitosan, a low-molecular weight product dissolved in an acetic acid solution was prepared. Grafted polymers were synthesized using a microwave digester. Results showed that TEMPO-mediated oxidation as the cellulose pretreatment is a key factor to obtain the grafted polymer CNF-g-CH. A reaction mechanism has been proposed where the amino group of chitosan attacks the carboxylic group of oxidized cellulose, since non-oxidized CMFs do not achieve the desired grafting. ^13^C NMR spectra, elemental analysis and SEM images validated the proposed mechanism. Finally, CNF-g-CH was used as a promising material to remove water-based inks and dyes from wastewater.

## 1. Introduction

In recent years, intense efforts have been made to produce new eco-friendly polymeric materials from renewable sources, more biodegradable, biocompatible and capable to replace conventional synthetic polymers [1,2,3]. These facts have stimulated the interest in abundant natural biodegradable polymers such as cellulose and chitin [4].

Cellulose is a polysaccharide, isolated by Anselme Payen in 1838 [5]. Its structure is formed by repeated β-d-glucopyranose molecules linked by covalent bonds between the equatorial OH groups of C4 and C1 carbon atoms [6]. In addition, cellulose is the most abundant, renewable, biodegradable natural polymer on the Earth, being the major structural component of the cell walls of plants. Furthermore, it is also generated as an exopolymer by some microorganisms [7,8,9,10]. Cellulose has a large number of applications in several fields, such as biomedicine, composites, paper industry, packaging, membranes, food or pharmacy, due to its low cost [11].

Cellulose nanofibers (CNFs) and cellulose microfibers (CMFs) were first obtained by Turbak et al. in 1983 [12] as a gel product prepared by a mechanical treatment of wood cellulose pulps using a homogenizer at high temperature and pressure. CNFs and CMFs are flexible fibers, of which dimensions depend on the used raw material, such as wood species (eucalyptus, pine, and birch), agroforestry wastes (sugar, rice, corn, potato, and wheat) or bacteria; and the methods used for their production [11,13,14,15,16].

Chitosan (CH) is a linear copolymer of n-acetyl-D-glucosamine and D-glucosamine, obtained from the deacetylation of chitin, the second most abundant natural polymer [17,18,19]. When deacetylation of chitin is greater than 60%, it is called CH [20], and it has interesting properties, such as its biocompatibility, biodegradability, bioresorbable, nontoxic, and antibacterial properties [21,22,23,24]. CH is soluble in acid solutions, being the most commonly used acetic acid solution. However, it is insoluble in organic solvents and aqueous solutions at neutral and basic pH [20].

**Figure 1:**
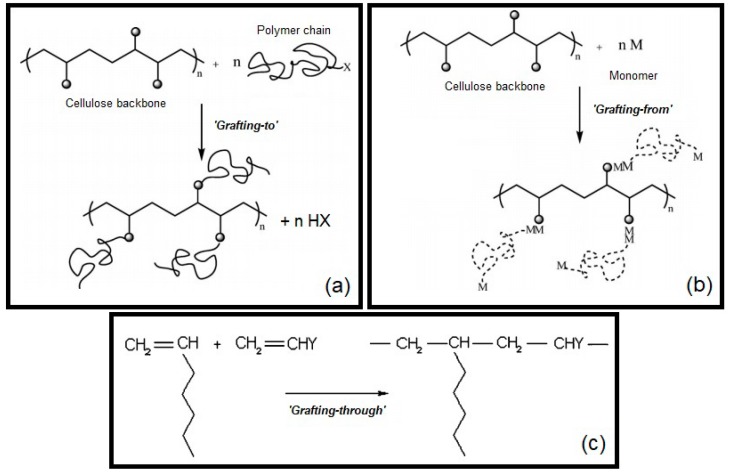
Schematic representation of grafting approaches: (**a**) “grafting-onto (or -to)”; (**b**) “grafting-from”; (**c**) “grafting-through”. Adapted from Roy et al. [29], with permission from The Royal Society of Chemistry, 2009.

The studies to improve the properties of natural polymers have increased significantly in the last few years, with the aim of both improving their performance for different applications and finding new biopolymers to replace synthetic polymers. An effective method to improve the polymer properties or to obtain a product with new properties is chemical modification through graft copolymerization. This technique involves the grafting of a long chain of a homopolymer as a backbone onto the surface of another [25,26,27,28]. Grafting is carried out by different methods that can be classified in three major groups: ‘‘grafting-onto (or -to)’’, ‘‘grafting-from’’ and ‘‘grafting-through” [25,29]. In the “grafting-onto” approach, an end functional group of a polymer reacts with the functional groups that are located on the polymer backbone. Generally, this mechanism has low reaction efficiency due to the low activity of macromolecular reactions, limited by the crowding of chains at the surface [10,29,30,31]. The “grafting-from” approach is the most commonly used technique, where the growth of polymer chains occurs, mainly via free radicals, from initiating sites on the backbone. One of the advantages of this method is the high graft density achieved due to the easy access of the reactive groups to the chain ends of the polymer. Reactive groups along the main chain can be created by irradiation or chemical treatment, followed by the addition of a monomer/polymer [10,29]. Finally, in the “grafting-through” approach, a macromonomer, usually a vinyl macromonomer, is copolymerized with a low-molecular weight comonomer [10,25,29]. A schematic representation of these three grafting approaches, using cellulose as the backbone, is shown in Figure 1.

The reactions are based on different synthesis routes: (a) free-radical polymerization; (b) ionic and ring-opening polymerization; and (c) living radical polymerization [29]. Free-radical polymerization is employed in 60% of copolymerization reactions. The process consists on a chain reaction with three steps: initiation, propagation and termination [30,32]. Ionic and ring-opening polymerization requires strict reaction conditions, and consequently, few studies have been undertaken [29]. An alternative approach is the graft of well-defined polymers onto another polymer, in which a macromonomer is firstly preobtained by anionic or cationic polymerization, and then it is coupled to the backbone polymer via an SN2 nucleophilic reaction [33]. Finally, living radical polymerization is defined as a chain growth process without chain-breaking reactions, in which there is not a termination step [34].

Cellulose grafting has been developed with natural and synthetic monomers and polymers. Roy et al. and Kang et al. [10,29] reviewed several grafting processes involving cellulose matrices and different monomers and polymers. They concluded that the most commonly used route employs monomers with double carbon bonds via radical polymerization. For CNFs, Littunen et al. [35] used a free-radical polymerization with acrylates and methacrylates. On the other hand, CH as a backbone was used in several graft polymerizations via free radicals using polymer chains, such as poly N-isopropylacrylamide, acrylic acid or polystyrene [36]. However, the use of cellulose and CH as copolymers together has been briefly studied. Only Arsad et al. have published the use of a nanocellulose-graft-chitosan (CNF-g-CH) polymer for the removal of ethyl orange dye from wastewater [18,37]. They used a methodology based on Ghosh et al. [38] grafting a polysaccharide, similar to cellulose, and a polyacrylamide. However, in our research, cellulose and CH have not double bonds as polyacrylamide to develop a free-radical polymerization. Therefore, the reaction mechanism is still unknown.

In this research, CNF-g-CH and microcellulose-graft-chitosan (CMF-g-CH) polymers have been studied in detailed to assess the reaction mechanism. Recycled cellulose pulp was used as the raw material without any pretreatment, to produce CMFs; and with a NaClO/NaBr/2,2,6,6-tetramethylpiperidin-1-oxyl-radical (TEMPO)-mediated oxidation pretreatment, to obtain CNFs. Finally, a promising application of the new polymer was proved to remove water-based inks (copper phthalocyanine blue and carbon black pigments) and dyes (methyl orange) from wastewater as an alternative to use separately CNFs and CH.

## 2. Materials and Methods

**Figure 2:**
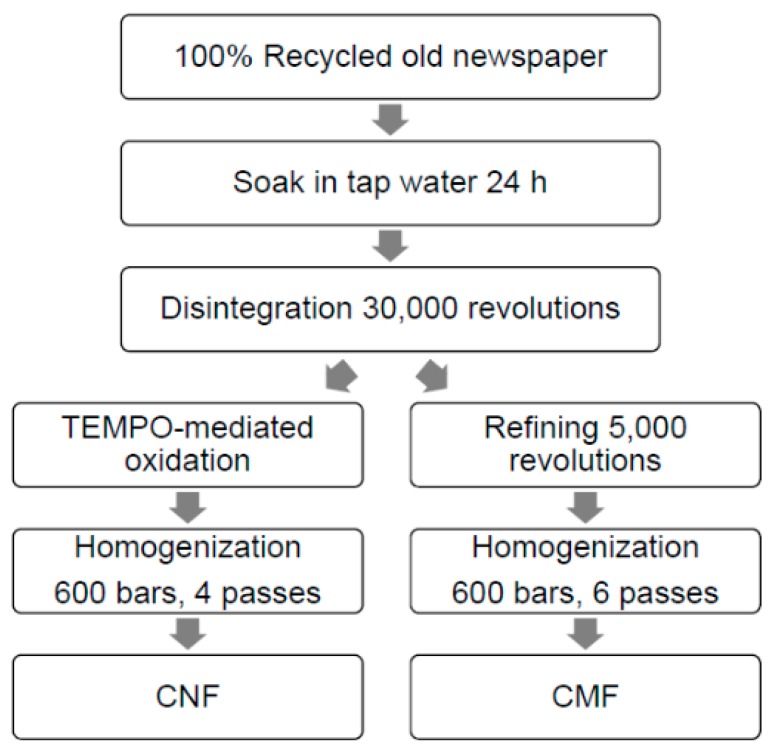
CMF and CNF preparation.

CMFs and CNFs were obtained from 100% recycled old newspaper (ONP) with 14% ash content, manufactured by Holmen Paper Madrid (Madrid, Spain). Figure 2 shows a scheme of CMF and CNF production. First, ONP was left to soak in water to encourage the swelling of fibers. Then, it was disintegrated by using a pulp disintegrator (PTI Paper Testing Instrument GmbH, Vorchdorf, Austria) at 30,000 revolutions and a 3.0 wt. % consistency, according to ISO 5263-1 standard [39]. CMFs were obtained by refining the pulp in a PFI mill, a laboratory-scale refiner used to beat pulp fibers manufactured by Hamjern Maskin AS (Hamar, Norway). Once the pulp was refined, it was diluted to a 1.0 wt. % consistency and six steps of homogenization at 600 bars were applied in a laboratory homogenizer PANDA PLUS 2000 manufactured by GEA Niro Soavy (Parma, Italy). CNFs were obtained using a common chemical pretreatment with TEMPO at 25 °C and pH 10 (TEMPO-mediated oxidation), according to Saito et al. [40], with 10 mmol of NaClO per gram of dry pulp. When TEMPO-mediated oxidation was applied to cellulose, C6 primary hydroxyls of cellulose were expected to be oxidized to C6 carboxylic groups, which facilitated the fibrillation of the cellulose due to repulsive forces. Once the pulp was oxidized, a filtration cleaning process was performed using distilled water until the pH was 7, to remove all the reaction medium reagents. Finally, four steps of homogenization at 600 bars were applied in a laboratory homogenizer.

According to Berglund [41], both CMF and CNF suspensions were stored at low concentrations (less than 2 wt. %) to avoid their aggregation. They were refrigerated at 4 °C, after adding some drops of bactericide (5 drops/L) to prevent the bacterial growth, until they were used. CMFs and CNFs were characterized according to Balea et al. [42] and their properties are listed in Table 1. All characterization parameters of CMFs and CNFs were carried out by duplicate and the average error between replicates was always under 5%.

**Figure 3:**
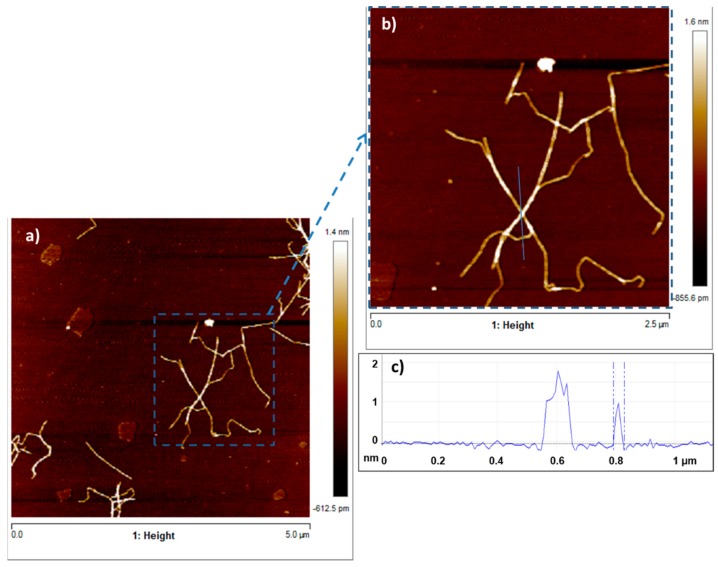
AFM images of CNFs (**a**,**b**) and depth distribution of a cross section of the AFM image (**c**).

CNFs produced by TEMPO-mediated oxidation have a higher content of carboxylic groups (0.79 mmol COOH/g) than CMFs (0.07 mmol COOH/g) produced only by mechanical processes, without any chemical pretreatment. CNFs with a higher content of carboxylic groups have a lower degree of polymerization than CMFs, which agrees with the apparently shorter nanofibrils observed in the Atomic Force Microscopy (AFM) analysis of CNFs (Figure 3). Moreover, the presence of NaClO as an oxidant in TEMPO-mediated oxidation reduces the amount of lignin, producing a CNF suspension with a low kappa number compared to the amount of lignin of a CMF suspension. Additionally, these new charged carboxylic groups after TEMPO-mediated oxidation produce repulsive forces that help the defibrillation of cellulose during the mechanical process, obtaining a nanofibrillated suspension with high nanofibrillation yield (the nanofibrillation degree of CNFs was 80% compared to that of CMFs (39%)), meaning that almost all the solid material is effectively nanosized. The cationic demand represents the anionic nature of the fibers, and it has been traditionally used to determine the extent of fiber delamination of beaten papermaking pulps. High cationic demand is expected for CNFs due to large fibrillation and the anionic nature of cellulosic materials suspended in water [11,12,42].

**Figure 4:**
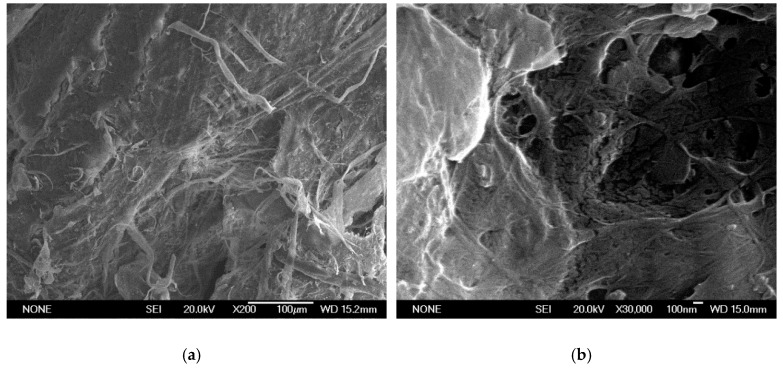
SEM images of CNFs. (**a**): ×200 magnification; (**b**): ×30,000 magnification.

Figure 4 shows the SEM images of the CMF produced only by mechanical processes. Several images were taken with different resolutions, in which the length and thickness of CMF were analyzed. The fiber diameter of CMF is between 100 nm and 5 µm, whereas its length varies from 1 µm to 300 µm. Figure 3a,b show the AFM images of the CNF produced from the recycled pulp by TEMPO-mediated oxidation, followed by mechanical homogenization. The analysis of the images shows that the length of the nanofibers varies from 0.5 µm to 2.5 µm and their thickness ranges from 40 nm to 100 nm. Some of the nanofibers are not elemental because they contain several elemental fibrils, as shown in Figure 4. These nanofibers have a larger thickness, around 100 nm. The height is always below 2 nm, with some exceptions due to impurities, as shown in Figure 3c.

Low-molecular weight CH, supplied by Sigma-Aldrich (CAS: 9012-76-4, San Luis, MO, USA), was prepared at 1 wt. %. Chitin is highly insoluble with low chemical reactivity. However, CH has some unique characteristics that make it more applicable than chitin, including its availability in different physical forms. However, CH is insoluble in either organic solvents or water with pH above 6.5, but under these conditions, it is soluble in most aqueous acids [43]. For that, CH was dissolved in a 1 vol. % of acetic acid solution. The CH solution was stirred until CH was fully dissolved (pH = 4.0–4.5) and stored at 4 °C.

Synthesis of grafted polymers based on nanocellulose and microcellulose were carried out by using a microwave digester as a heating source. According to HPS et al. [44], CH and cellulose materials should be dissolved prior to preparation of blends. In this method, a solution of 0.1 wt. % CH in acetic acid was mixed with an aqueous suspension of CMFs or CNFs (0.1 wt. %). Then, blends were stirred for 10 min under strong agitation (1000 rpm) to ensure that they were well dispersed before the reaction occurred. Afterwards, the mixtures of both polymer solutions (pH = 4.5–5.0) were heated in three reaction vessels of 50 mL to accelerate the reaction, using the microwave digester ETHOS Easy manufactured by Milestone SRL (Sorisole, Italy). First, a temperature ramp for one minute was applied until the temperature reached 70 °C, and then the temperature was maintained for 15 min. After that, reaction vessels were cooled and undisturbed for 24 h. Finally, solutions were washed with acetone to facilitate precipitation of grafted polymers in an acetone–sample ratio of 1:4 [18] and centrifuged in a Sigma 3-16P centrifuge (Osterode am Harz, Germany) at 5000 rpm. Nongrafted CH and nanocellulose remained in the supernatant, whereas the grafted polymer precipitated in the vessels as a gel phase. Finally, gel grafting product was dried at room temperature and powdered in an agate mortar to characterize it.

Grafted polymers were characterized using three methods: (a) ^13^C NMR spectra of solid samples of CH, CMFs, CNFs, CMF-g-CH and CNF-g-CH were recorded at 400 MHz Wide Bore with a Bruker AV spectrophotometer (Bruker Corp., Billerica, MA, USA). Carbon bonds were analyzed to assess the grafting reaction by studying the spectra, which shows the peaks intensity of carbon bonds versus chemical shift (δ, ppm); (b) elemental analyses of CH, CMFs, CNFs, and copolymers grafted were undertaken with an elemental analyzer LECO CHNS-932 (LECO Corp., Saint Joseph, MI, USA) to determine carbon, nitrogen and hydrogen contents of the samples. Elementary mass balances were carried out to determine the chitin and CH contents in commercial CH samples, cellulose (oxidized and non-oxidized) and the lignin content in CMF and CNF samples. Additionally, the elementary mass balances were done to determine CH, nanocellulose and microcellulose contents in the grafted polymers produced; and (c) scanning electron microscopy (SEM) was used to study CH, CMFs, CNFs and grafted polymer morphologies at different magnifications using a JEOL JSM 6335F microscope (JEOL, Tokyo, Japan). The samples were prepared using the following procedure: (a) The surface of the sample holder of the equipment was covered with a nonconductive tape; (b) a diluted solution of CH, CNFs, CMFs or grafted polymer samples was distributed on this tape and the sample holder was placed in an oven at 60 °C; and (d) the sample holder was placed inside a sputter instrument, where it was coated with a Au/Pd layer (≈ 15 nm thick).

To study the efficiency of the grafted polymer to remove of water-based inks and dyes from wastewater, three synthetic solutions were prepared using two-model water-based inks from copper phthalocyanine blue (blue ink) and carbon black pigments (black ink), and methyl orange as a dye (orange dye). Table 2 shows the molecular structures, maximum wavelengths and concentrations of the models used. The optimal grafted polymer was used for this study. Ten milliliters of synthetic wastewater was added in laboratory test tubes. Then, different dosages of solid- and gel-grafted polymer were added and the mixture was shaken strongly for 30 s. Samples were prepared by duplicate. Immediately, absorbance was measured in all samples and the ink/dye removal was calculated.

## 3. Results and Discussion

### 3.1. Grafting Methodology

To validate the graft of polymers, CH, CMFs, CNFs and both grafted polymers were analyzed by ^13^C NMR to determine the carbon bonds. Additionally, elementary mass balances based on elementary analysis were carried out. In case of CMFs and CNFs, the kappa number and carboxylic groups were also used to determine lignin content and the amount of cellulose oxidized. Table 3 and Table 4 show the composition of commercial CH, CMFs and CNFs.

**Figure 5:**
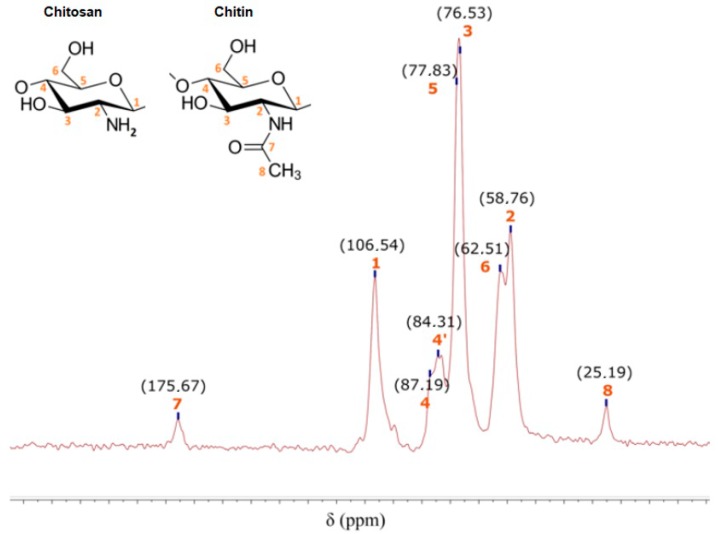
^13^C NMR spectrum of commercial chitosan.

Figure 5 shows commercial CH spectrum and the carbon numbers of CH and chitin molecules. Peaks between 58.76 ppm and 106.54 ppm belong to the CH and chitin ring; among them, the C4 peak is divided into two peaks, 87.19 ppm and 84.31 ppm, belonging to crystalline and amorphous shapes, respectively. The C7 and C8 peaks belong to the chitin molecule that has not been deacetylated.

**Figure 6:**
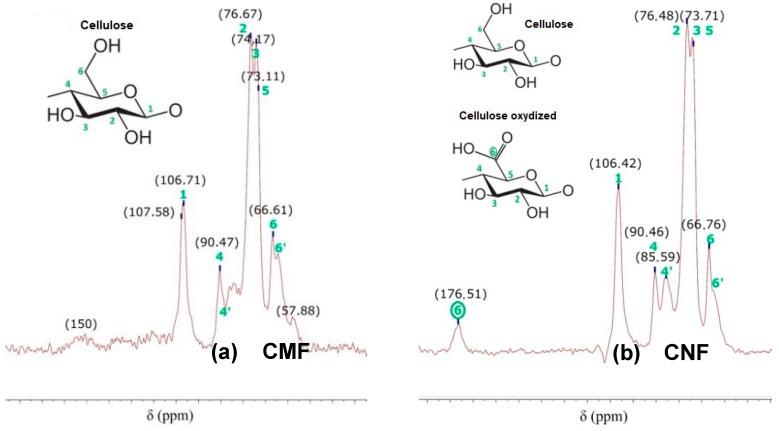
^13^C NMR spectra of (**a**) CMF and (**b**) CNF.

Figure 6 shows ^13^C NMR spectra of CMF and CNF. According to Table 1 and Table 4, CMFs have hydroxyl groups at the C6 position and a significant content of lignin. This fact is shown in Figure 6a, in which there are two peaks at 57.88 and 150 ppm attributed to lignin. However, in the CNF, a part of the hydroxyl groups at the C6 position were converted into charged carboxylic groups, due to the TEMPO-mediated oxidation pretreatment. Additionally, CNFs present a low content of lignin. The CNF spectrum shows a peak at 176.51 ppm (represented by ⑥ in Figure 6b), indicating the carboxyl bond due to the TEMPO-mediated oxidation of cellulose. In general, the other peaks of CMF and CNF are the same, as shown in Figure 6. In both cases, C4 and C6 carbons have two peaks because of crystalline and amorphous phases. Finally, the same order and chemical shift of cellulose- and CH-ring carbons can be observed, except for C2 carbon, in which the amino group of CH is linked to the hydroxyl group at the C6 position of cellulose oxidation.

**Figure 7:**
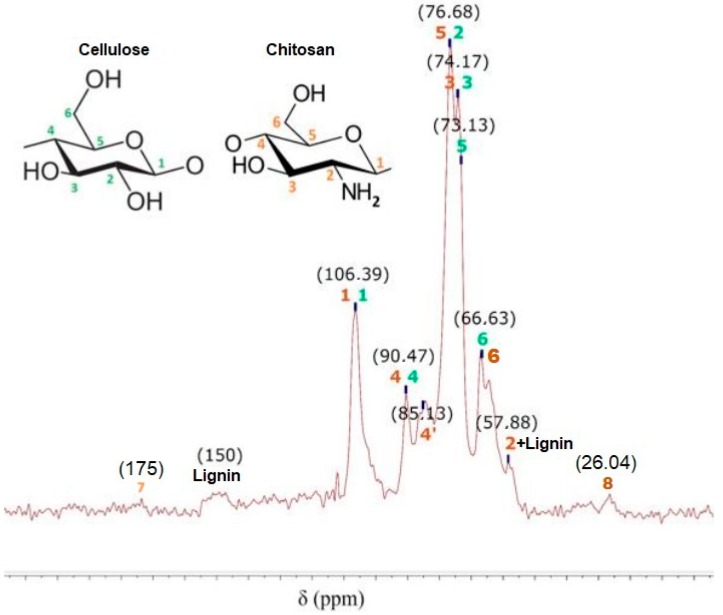
^13^C NMR spectrum of CMF-g-CH after copolymerization.

The NMR spectrum of CMF-g-CH is shown in Figure 7. CMF-g-CH has the similar peaks as the CMF; nevertheless, bonds associated exclusively to CH decrease their peaks such as the C2 and C4 peaks, and the C7 and C8 peaks from chitin bonds almost disappear. In addition, new bonds are not shown in the spectrum. The results indicated a small amount of CH linked to the CMF and the removal of CH in the centrifugation step. This fact is verified when elemental analysis of CMF-g-CH and elementary mass balances were done. CH only represents 3.8% of the obtained polymer, which indicates the final polymer is a nongrafted product.

**Figure 8:**
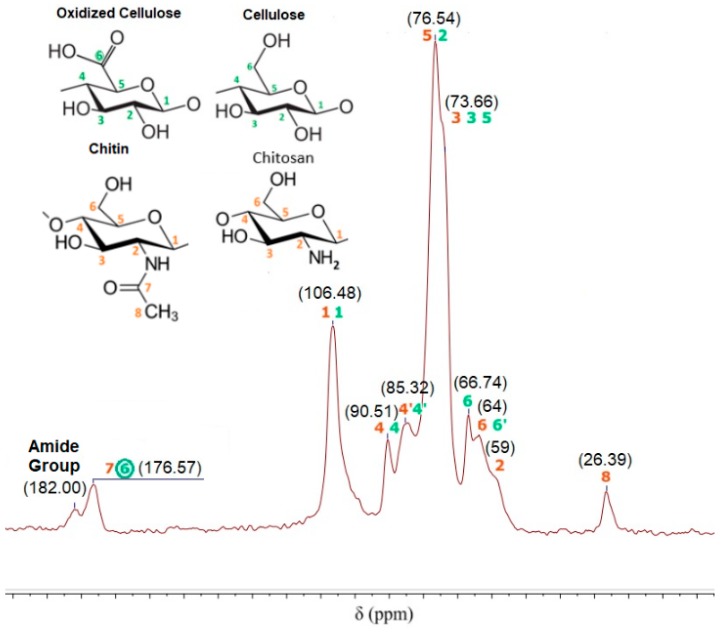
^13^C NMR spectrum of CNF-g-CH after copolymerization.

On the other hand, the ^13^C NMR spectrum of CNF-g-CH is shown in Figure 8. Grafted polymer has the peaks corresponding to the native cellulose, oxidized cellulose, CH and chitin. The peak at 176.57 ppm indicates a carboxylic bond formed in the TEMPO-mediated oxidization of cellulose and the carboxylic bond of non-deacetylated chitin. Additionally, the absence of peaks at 57.88 and 150 ppm indicates no lignin content in the grafted polymer. In this case, the peaks from CH and chitin are maintained in the spectrum. The main characteristic of the spectrum is a new bond at 182.00 ppm that indicates the presence of an amide group in the sample. Furthermore, elemental analysis of CNF-g-CH and elementary mass balances of carbon and nitrogen indicate a CH content of 16% in the grafted polymer.

**Figure 9:**
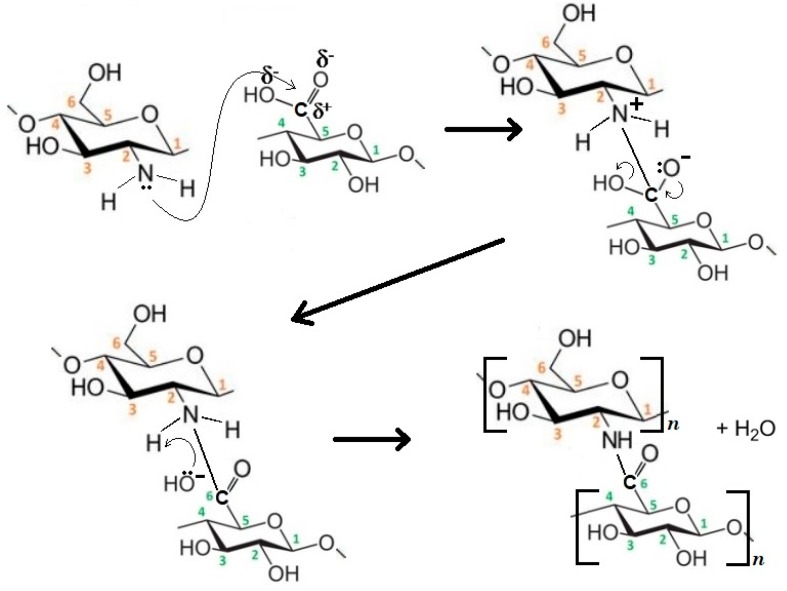
Copolymerization reaction mechanism of CNF-g-CH.

The main difference between the CMF and CNF used in the grafted polymers is the carboxylic group presented at the C6 position of CNF with high reaction capacity. This carboxylic group is capable to react with amino groups of CH in the presence of heat. In this case, a “grafting to (or onto)” approach could be the most appropriate mechanism, in which a nucleophilic substitution reaction is developed due to heat. Figure 9 explains the reaction mechanism in three steps. In the first one, the reaction is initiated with microwave heat, in which the nucleophilic group attacks the carboxylic group. Then, the leaving group (OH^−^) departs with an electron pair and carboxylic bond is formed in the principal molecule. Finally, a water molecule is formed. Although side reactions of the chemical medium used to produce the CNF could take place during graft copolymerization, their impact is not noticeable because almost all TEMPO-oxidation reagents were removed after washing. Recently Yang et al. [45] have developed a novel biopolymer-based aerogel produced by freeze-drying a hydrogel, synthesized from cross-linking bifunctional hairy nanocrystalline cellulose and carboxymethylated CH. They showed that hairy nanocristally cellulose, bearing aldehyde and carboxylic acid groups, facilitated the cross-linking with CH through imine bond formation, while providing negatively charged functional groups.

**Figure 10:**
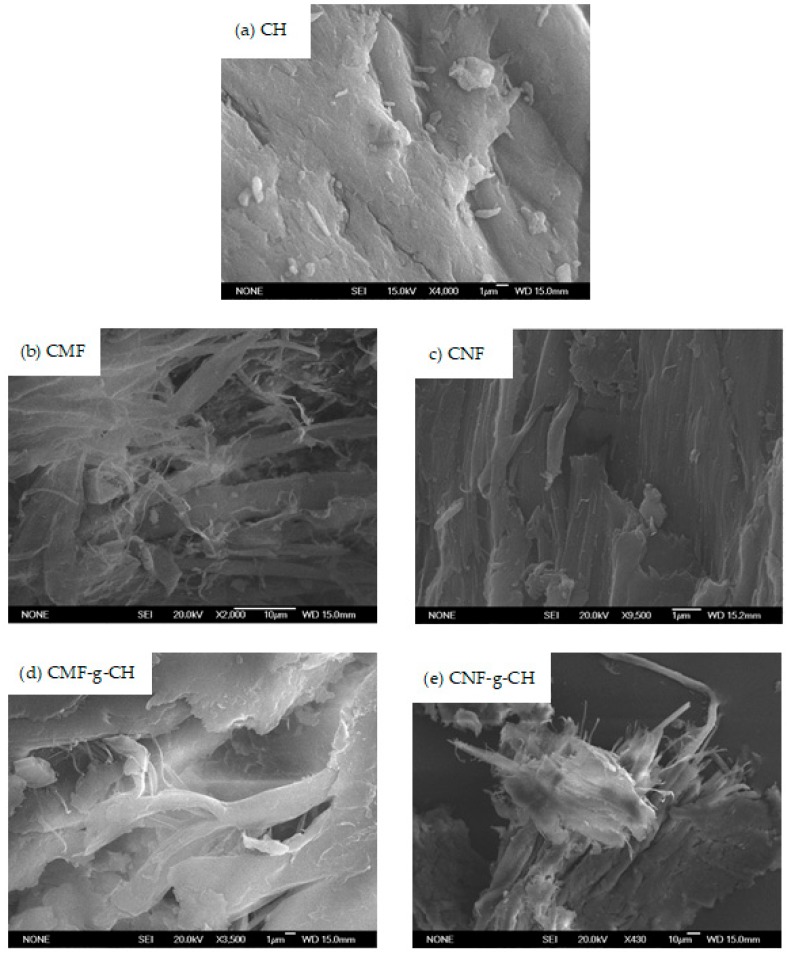
SEM Images: (**a**) CH; (**b**) CMF; (**c**) CNF; (**d**) CMF-g-CH; (**e**) CNF-g-CH.

SEM images (Figure 10) show, at different magnifications, the raw materials (CH, CMF and CNF) and the grafted copolymers (CMF-g-CH and CNF-g-CH).

### 3.2. CNF-g-CH Applications

Water-based inks are not removed by traditional treatments and the recyclability of printed papers with these kinds of inks is limited. In a previous paper, we have shown the viability to remove them by a treatment based on a dual system with CNFs and a cationic polyacrylamide (cPAM). The results show that, firstly, adsorption onto TEMPO-oxidized CNFs occurs and, secondly, a flocculation process with a synthetic cPAM produces the decolorization of the solution [46]. According to Balea et al. [46], the separate addition of the additives and the addition order highly affect the ink removal behavior. Based on the Jar test methodology, the addition of cPAM before the CNF is less effective than the addition of cPAM after the nanofibers [46]. On the other hand, the blend of cPAM and CNFs is not effective.

The aim of this research is to simplify the proposed treatment by developing a product capable of removing flexographic inks in only one step and in a short time. Moreover, the use of a grafted CNF polymer, prepared with two natural polymers from renewable natural resources, has many advantages such as its biodegradability.

**Figure 11:**
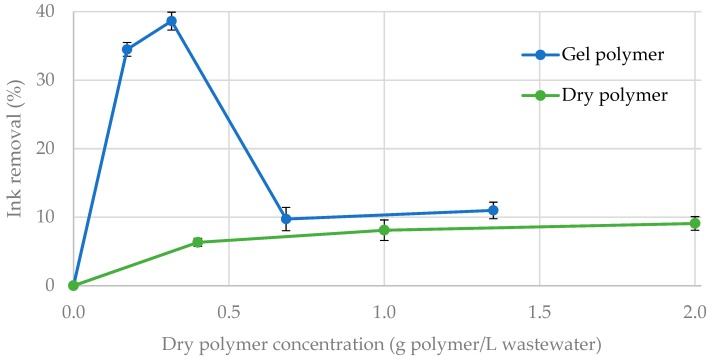
Blue ink removal with CNF-g-CH.

**Figure 12:**
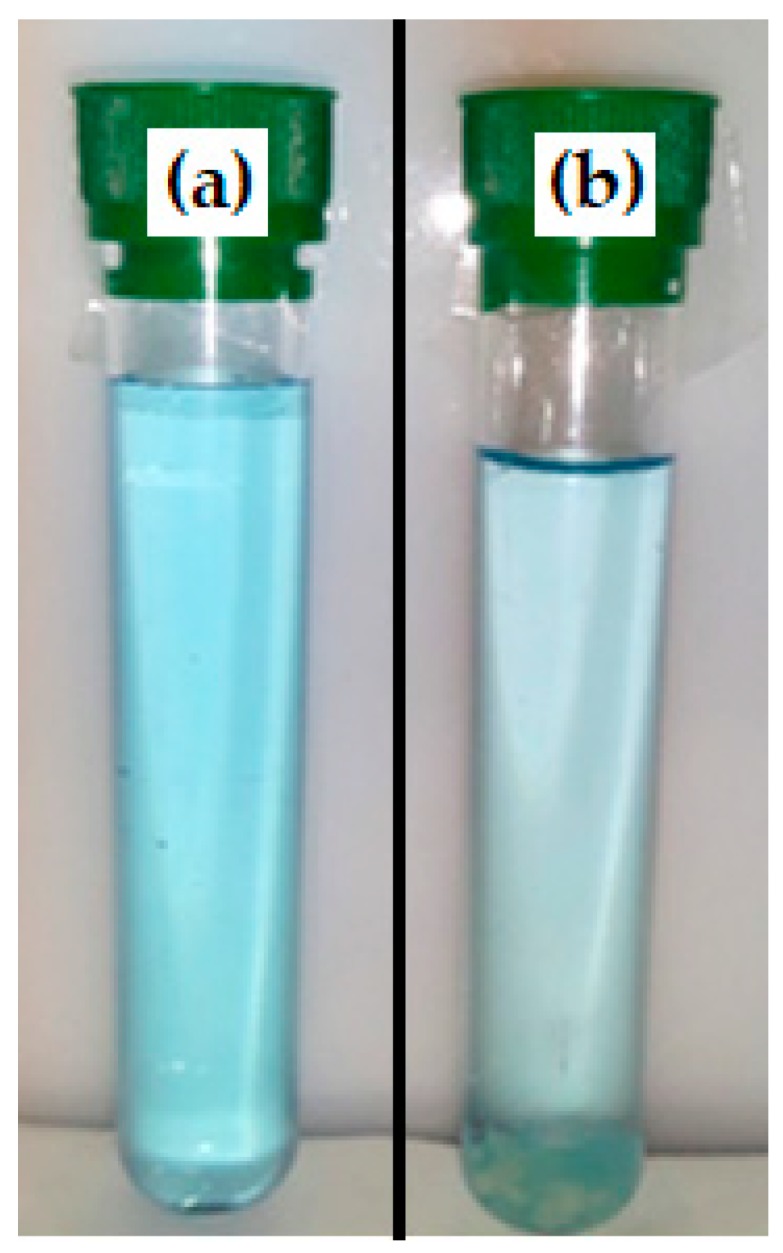
Blue ink removal: (**a**) blank; (**b**) 30 s after the gel polymer application.

Therefore, preliminary experiments were carried out by duplicate to evaluate the efficiency of the CNF-g-CH to remove water-based inks and dyes from synthetic wastewater. The grafted polymer with 16% CH and 84% CNF was applied in solid and in gel phase (1.7% of dry polymer). Results show a higher ink removal when gel polymer was applied compared to the dried polymer. This fact is due to higher adsorption capacity of the gel polymer, which may be improved in the dry polymer due to the partial hornification of the nanofibers during drying. Therefore, when CNF-g-CH was used as solid, only some blue particles were disposed on the surface of the laminated dry product. In both cases, the ink removal was measured after the samples were shaken (Figure 11). In these conditions, gel polymer produces an ink removal of 40% using 0.32 g of dry polymer per liter of synthetic wastewater (Figure 12). According to Balea et al. [46], ink would be adsorbed by the presence of CNF, and CH would be the responsible for the flocculation step. If contact time is increased from 30 s to several hours, a total ink removal (>95% color) is obtained.

**Figure 13:**
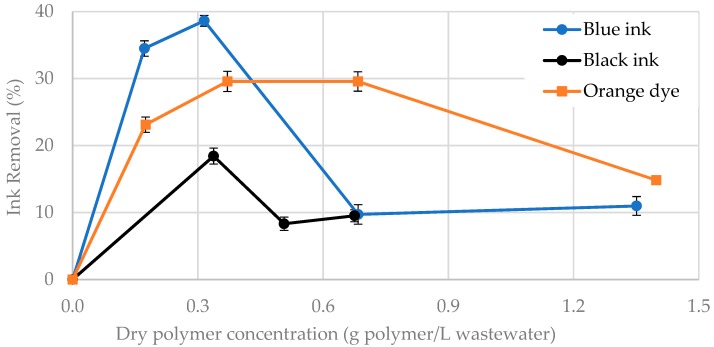
Ink and dye removal with CNF-g-CH in gel phase.

The gel grafted polymer was also used to remove the black ink and orange dye due to its efficiency in these conditions. As shown in Figure 13, the highest ink/dye removal is reached for 0.3–0.4 g of dry grafted polymer per liter of wastewater. The lower ink removal of blank ink compared to the blue one is according to the results obtained by Balea et al. [45]. The concentration of the gel-grafted polymer that needed to achieve the highest ink removal was the same for both inks. However, when CNFs and polyacrylamide were used separately as a dual-treatment [46], the optimal CNF concentration was the same for both inks (0.01 wt. %), but the polyacrylamide dose was higher for blank ink (0.01 wt. %) than for blue ink (0.0075 wt. %).

Although these experiments are very promising, a detailed research should be carried out to optimize the grafting conditions (CNF–CH ratio or microwave operation parameters) and their impact on water-based inks and dye removal.

## 4. Conclusions

Reaction mechanism based on a nucleophilic substitution reaction was proposed to graft CNFs and CH. TEMPO-mediated oxidation is a key factor to obtain the grafted polymer, where the nucleophilic group of CH (amino group) attacks the carboxylic group of oxidized cellulose. Based on this reaction mechanism, a promising application of CNF-g-CH was proposed, which simplified the available alternatives. The grafted polymer in the gel form was more effective to remove water-based inks and dyes. However, a detailed research is required to improve the effectiveness of the grafted copolymers in wastewater treatments and to optimize the reaction and treatment conditions.

## Figures and Tables

**Table 1:** Characterization of CMFs and CNFs.

Parameter	Unit	CMF	CNF
Concentration	(% by weight)	1.08	0.94
Nanofibrillation degree	(%)	39	82
Carboxylic groups	(mmol COOH/g)	0.07	0.79
Cationic demand	(meq/g)	0.04	0.46
Kappa number	(-)	135.9	31.2
Polymerization degree	(monomeric units)	703	235
Transmittance 400 nm	(%)	1.8	12.2
Transmittance 800 nm	(%)	8.7	27.6

**Table 2:** Water-based ink and dye characterization.

Name (Nomenclature in the Text)	Copper Phthalocyanine Blue (Blue Ink)	Carbon Black (Black Ink)	Methyl Orange (Orange Dye)
**Core structure**	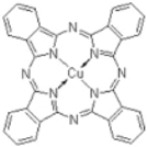	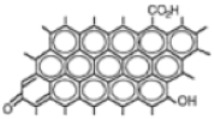	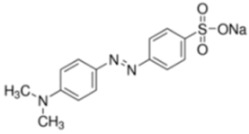
**Maximum wavelength (λ_max_), nm**	615	500	460
**Concentration, ppm**	4.5	2	4.5

**Table 3:** Commercial chitosan composition.

Compound	Commercial Chitosan (%)	Dry Commercial Chitosan (%)
**Chitosan**	72.7	81.6
**Chitin**	16.3	18.4
**Water**	11.0	-

**Table 4:** Dry CMF and CNF composition.

Compound	Dry CMF (%)	Dry CNF (%)
**Cellulose**	77.4	81.8
**Oxidized cellulose**	1.2	13.0
**Lignin**	21.4	5.2
